# Placental damages in preeclampsia – from ultrasound images to histopathological findings

**Published:** 2015

**Authors:** CG Predoi, C Grigoriu, R Vladescu, AE Mihart

**Affiliations:** * ”Carol Davila” University of Medicine and Pharmacy Bucharest, Romania; ** Obstetrics and Gynecology Department, University Emergency Hospital Bucharest, Romania

**Keywords:** preeclampsia, placenta, intraplacental haematomas, cystic areas, echogenic cystic lesion

## Abstract

Preeclampsia is a unique pregnancy-related disease that affects 5-7% pregnancies worldwide. Placental architecture is modified in PE and eclampsia. Placental morphology and cellular arrangement are important for oxygen delivery from the mother to the fetus. Fetal growth and well-being after 20 weeks of gestation are dependent upon successful placental development. This, in turn, is achieved by an enhanced maternal blood supply to the placenta (normal uterine artery Doppler) and growth/ differentiation of the gas-exchanging placental villi. Conversely, pregnancy with severe placental insufficiency exhibits abnormalities both in uterine artery and in umbilical artery Doppler, and results in adverse perinatal outcome. The evaluation of placental functioning is possible nowadays through ultrasound examinations. Sonographic images associated with placental lesions include cystic areas, heterogeneous appearance of the placental mass, and thick or thin placentas. Sonographic evidence of destructive placental lesions is defined as the evolution of irregular cystic spaces with echogenic borders – the echogenic cystic lesions. Histological examinations of placenta may confirm these antepartum observations. Decidual vasculopathy and accelerated villous maturity are considered indicative of uteroplacental vascular insufficiency. Perivillous fibrin deposition and intervillous fibrin are considered indicative of intervillous coagulation. Detailed sonographic evaluation of the placenta and histopathological confirmation after birth are used to identify lesions associated with preeclampsia, intrauterine growth restriction and adverse short and long-term perinatal outcome, but the presence of cystic images in the placenta is not uniformly associated with adverse perinatal outcome. Combining Doppler studies with placental texture studies may lead to satisfactory results.

**Abbreviations:** PE = preeclampsia; IUGR = intrauterine growth restriction; PI = pulsatility index; RIH = rounded intraplacental haematomas; TV = trophoblastic villi; MRI = magnetic resonance imaging

## Introduction

Preeclampsia (PE) is a unique pregnancy-related disease that affects 5-7% pregnancies worldwide. It is associated with hypertension and proteinuria. Despite long years of research, the mechanisms underlying the cause and progression of PE remain poorly understood. Risk factors include maternal and paternal family history of PE, nulliparity, ethnicity and existing disorders with several vascular dysfunctions, hypertension or inflammation such as diabetes, chronic hypertension, obesity, kidney disease, systemic lupus erythematosus and antiphospholipid syndrome [**8**,**9**]. Because of lack of predictive early markers and effective pharmaceutical interventions, PE is a serious obstetric complication leading to increased maternal and fetal morbidity and mortality.

The primary cause of PE is the widespread apoptosis of cytotrophoblast cells. The invasion of uterine spiral arterioles by trophoblasts is limited to the superficial portions of the decidua, and 30-50% of these arterioles in the placental bed escape trophoblast remodeling. The mean luminal diameter of uterine spiral arterioles in women with PE is less than one-third of the diameter of similar vessels from uncomplicated pregnancies. The uteroplacental perfusion is as such reduced, and the placenta becomes ischemic as gestation progresses. This causes fetal hypoxia as well as morphological and histological changes in the placenta, leading to PE or PE-associated IUGR, which contributes to premature delivery and fetal death.

Placental architecture is modified in many maternal diseases such as PE and eclampsia. Placental morphology and cellular arrangement are important for oxygen delivery from the mother to the fetus. Fetal growth and well-being after 20 weeks of gestation are dependent upon successful placental development. This, in turn, is achieved by an enhanced maternal blood supply to the placenta (normal uterine artery Doppler) and growth/ differentiation of the gas-exchanging placental villi. Conversely, pregnancy with severe placental insufficiency exhibits abnormalities in both uterine artery and umbilical artery Doppler, and results in adverse perinatal outcome. The evaluation of placental functioning is possible nowadays through ultrasound examinations. Histological examinations of placenta may confirm these antepartum observations. Decidual vasculopathy and accelerated villous maturity are considered indicative of uteroplacental vascular insufficiency. Perivillous fibrin deposition and intervillous fibrin are considered indicative of intervillous coagulation.

## Ultrasound examination

Diagnostic procedures during pregnancy complicated with pregnancy-induced hypertension/ preeclampsia include clinical examinations (periodical blood pressure measuring), ultrasound examinations, laboratory testing, fetal well-being assessments. Detailed ultrasound examination of the fetus at 18–20 weeks also includes placenta-related information beyond the fetal measurements and morphology: determination of placental length and thickness, number of cord vessels, cord insertion, and the assessment of placental texture. Uterine artery Doppler evaluation is performed by color and pulsed Doppler mapping. Mean pulsatility index (PI) values > 1.45 or bilateral early diastolic notches are considered abnormal [**6**,**10**,**11**].

**Fig. 1:**
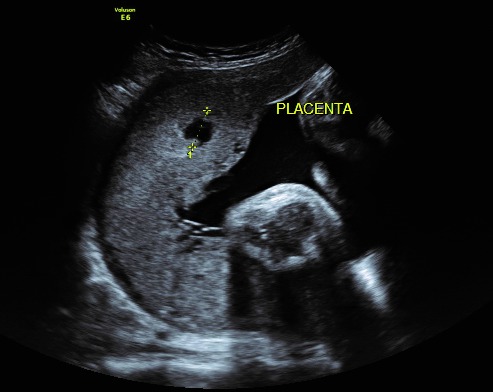
Echogenic cystic lesion

Placental texture may be first assessed at the 16-week examination and periodically until 36 weeks of gestation. Sonographic images associated with placental lesions include cystic areas, heterogeneous appearance of the placental mass, and thick or thin placentas. Sonographic evidence of destructive placental lesions is defined as the evolution of irregular cystic spaces with echogenic borders – the echogenic cystic lesions (**Fig. 1**). 

**Fig. 2:**
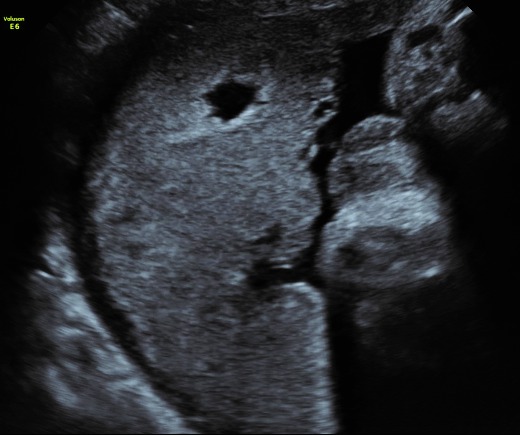
Placental infarction

Placental infarction hematoma has been associated with preeclampsia and intrauterine growth restriction (**Fig. 2**). 

The ultrasound appearance shows an echodense region inside an echolucent area (or a hypoechogenic image with a hyperechogenic rim), without demonstrable blood flow inside (a recently formed hematoma). Old hematomas within an infarcted area might not be identified by ultrasound, as they tend to appear echolucent; with time, a definitive diagnosis can only be made through histopathologic examinations [**12**-**14**]. 

A detailed sonographic evaluation of cysts includes an evaluation of shape, size and content as well as the absence/ presence of blood flow around or inside the cyst. Cystic areas are frequently observed in association with preeclampsia, growth restriction or fetal demise. Well-defined rounded cystic areas in the placenta are related to a higher risk of preeclampsia and intrauterine growth restriction. Several authors describe them as “rounded intraplacental haematomas” (RIH) and report that more than 50% of these cystic lesions were associated with placental infarcts reflecting maternal vascular underperfusion. Magnetic resonance imaging is also useful in diagnosing early placental insufficiency. Heparin is proposed as a treatment choice in preeclampsia with placental infarctions (**Fig. 2**) [**4**,**8**].

## Macroscopic study of placenta

Women with preeclampsia have placentas with reduced surface area, with a shape that is more oval than round, and an altered fetal/ placenta index [**3**]. 

**Fig. 4:**
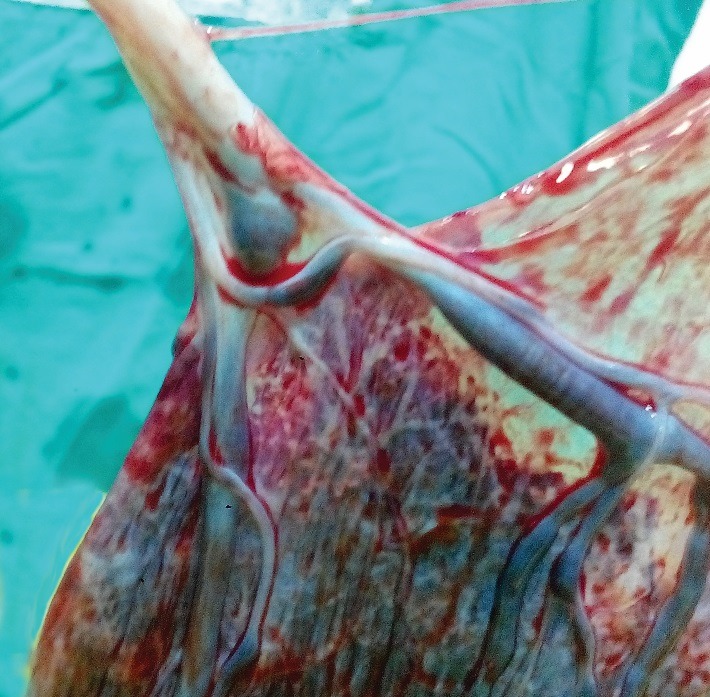
Velamentous cord insertion

Insertions of the umbilical cord into the placenta margin or into the fetal membranes (velamentous insertion) rather than into the main placental mass are associated with smaller placentas and smaller infants. Non-marginal, but markedly eccentric cord insertion associates with a weaker chorionic vascular distribution, inefficient transport gradient and a reduced birthweight for a given placental weight (the more eccentric the umbilical cord insertion, independent of the placental shape, the less efficient is the placenta working) (**Fig. 4**) [**4**,**15**]. 

## Histopathological studies

The placental infarcts are mainly due to occlusion of spiral arteries by thrombus, strangulation of the placental villi due to increased perivillous or intervillous fibrin/ fibrinoid deposition and impairment of the fetal circulation due to fetal thrombotic vasculopathy. Placental infarcts can be documented in approximately 20% of uncomplicated pregnancies and in 70% and 40% of patients with severe and mild preeclampsia. Vinnars et al. reported that infarcts involving more than 5% of the placenta could be observed in 39% of patients with severe preeclampsia [**15**,**16**]. 

On gross placental morphometric study, the PE placentas have significantly lower mean placental weights, thicknesses, diameters, and surface areas than control groups. The fetoplacental index is significantly decreased in the PE groups than in the control groups [**5**,**16**]. 

The stem villi of the PE placentas show numerous arteriosclerotic blood vessels with endothelial degeneration presenting progressive fibrosis, stem villous perivasculitis, and subsequent lumen obliteration [**3**,**5**,**9**,**16**]. 

Perivillous fibrin and intervillous fibrin deposition, which also extend to the intervillous bridges are observed in PE placentas. The number and structure of trophoblastic villi (TV) specifically vary in PE. The total numbers of TV are significantly lesser, indicating distal villous hypoplasia [**1**-**3**]. 

Numerous avascular TV surround the arteriosclerotic stem villi, possibly reflecting failure of vascular organization (villitis). TV syncytiotrophoblasts invariably develop clusters and sprouts to form syncytial knots. Histomorphometric findings indicate that the PE placentas have less villous surface area and smaller diameters, whereas the density of the TV was significantly higher [**15**,**16**]. 

## Practical issues

**Fig. 3:**
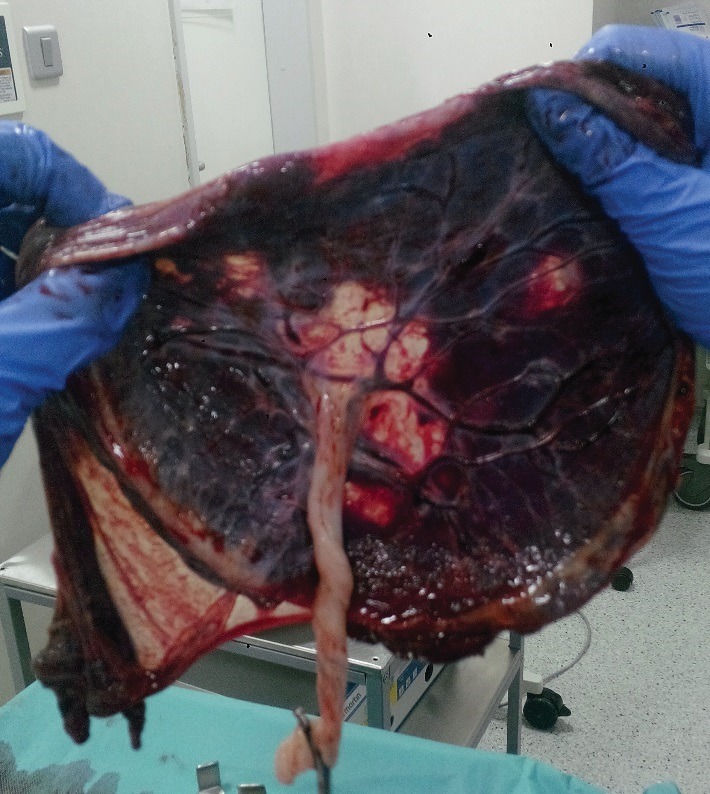
Avascular placental space

Nowadays, there is no treatment for placental infarction hematomas (**Fig. 3**). 

Several studies propose the use of low molecular-weight heparin and aspirin when placental lesions suggestive of infarcts are observed in the ultrasound scan. The studies suggest that the identification of placental lesions with ultrasound in the absence of fetal growth restriction may be managed by antithrombotic treatment. The association of fetal testing may more correctly identify the appropriate time for fetal birth, avoiding in utero fetal demise [**6**,**7**,**12**].

A detailed sonographic evaluation of the placenta and histopathological confirmation after birth are used to identify lesions associated with preeclampsia, intrauterine growth restriction and adverse short and long-term perinatal outcome, but the presence of cystic images in the placenta is not uniformly associated with adverse perinatal outcome [**10**]. Combining Doppler studies with placental texture studies may lead to satisfactory results. This is important for adequate referral to a tertiary perinatal center, for improved clinical outcome, both for mother and fetus [**1**,**4**,**6**,**13**].

## Conclusions

Pregnancies complicated by PE are reflected in the placenta both macroscopically and microscopically and may be diagnosed early by thorough placental serially ultrasound examinations. Although the placenta adapts well to the hypoxic condition in PE, the compensatory changes that occur are insufficient. These compensatory changes cause placental suboptimal development and placental dysfunction that leads to chronic fetal hypoxemia.
